# Supplementary material for: Genes adapt to outsmart gene-targeting strategies in mutant mouse strains by skipping exons to reinitiate transcription and translation
Source: Genome Biol. 2020 Jul 9;21:168. doi: 10.1186/s13059-020-02086-0 (PMC7350591; doi:10.1186/s13059-020-02086-0)

Supplementary Figure 1.

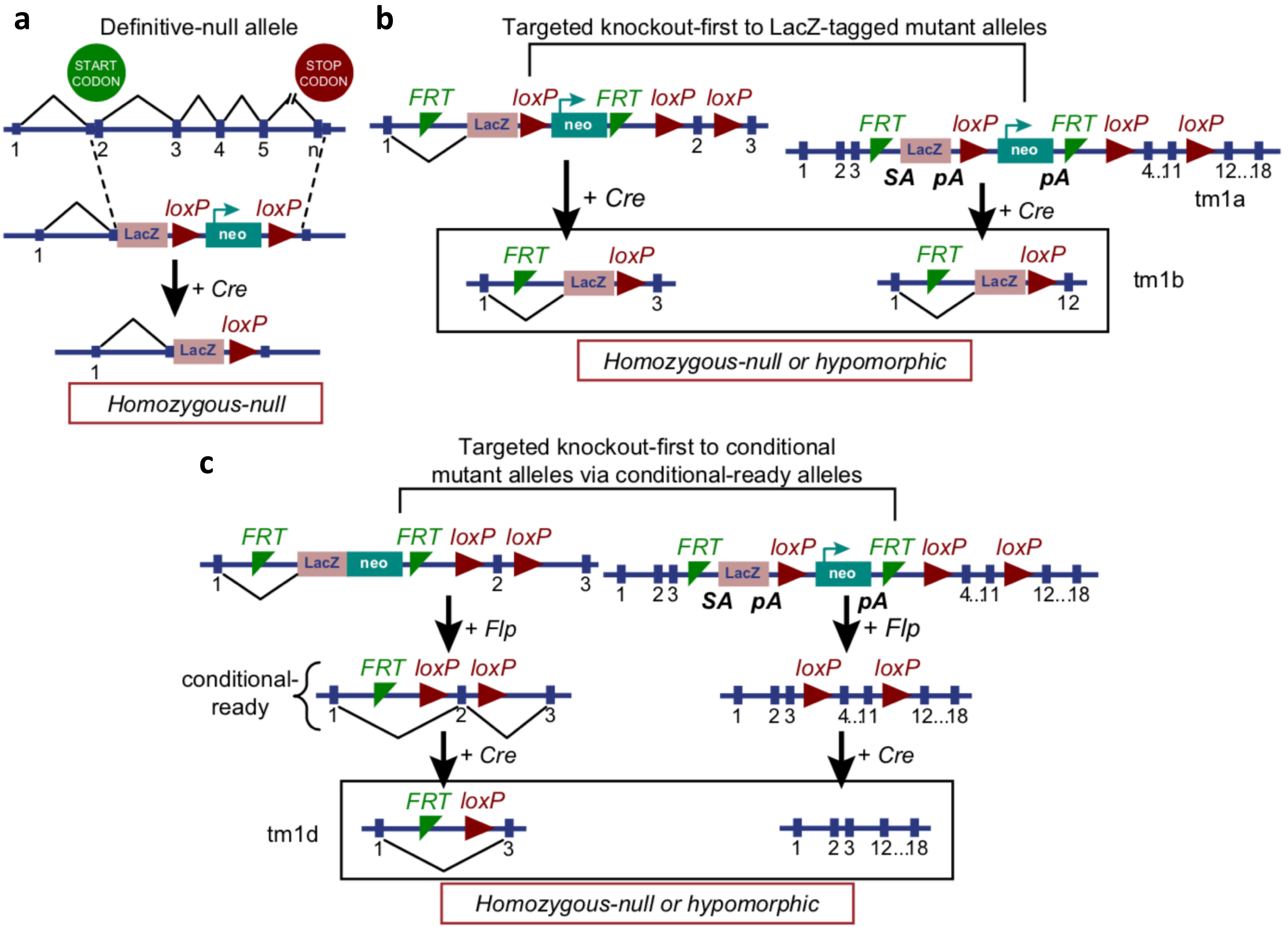

Supplementary Figure 2.

a

	***Rhbdf1*$^{+/+}$ *Rhbdf2*$^{-/-}$**	***Rhbdf1*$^{+/-}$ *Rhbdf2*$^{-/-}$**	***Rhbdf1*$^{-/-}$ *Rhbdf2*$^{-/-}$**
	Number (%) Expected 25%	Number (%) Expected 50%	Number (%) Expected 25%
e9.5	4 (29%)	8 (57%)	**2 (14%)**
e10.5	1 (4%)	18 (75%)	**5 (21%)**
e12.5	5 (42%)	6 (50%)	**1 (8%)**
e13.5	8 (40%)	9 (45%)	**3 (15%)**
e14.5	4 (44%)	4 (44%)	**1 (11%)**
e15.5	2 (25%)	1 (12.5%)	**5 (62.5%)**
e17.5	2 (33%)	2 (33%)	**2 (33%)**
e18.5	5 (33%)	9 (60%)	**1 (7%)**
P1-3	6 (23%)	16 (61%)	**4 (15%)**
>P4	9 (24%)	29 (76%)	**0**

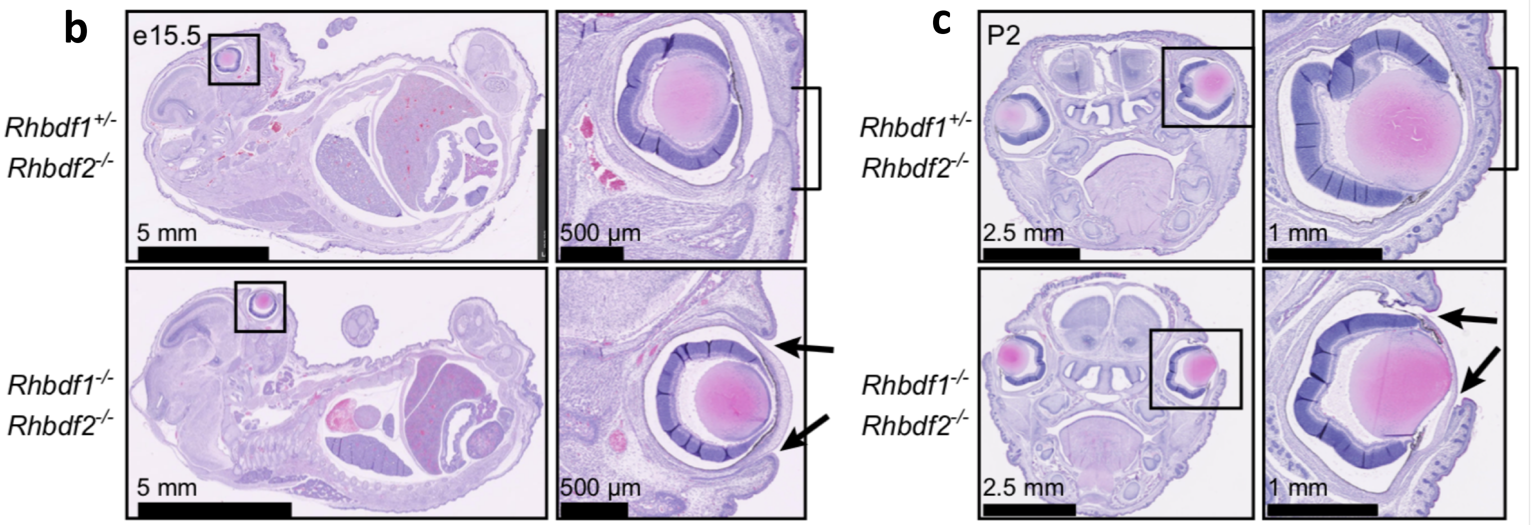

Supplementary Figure 3

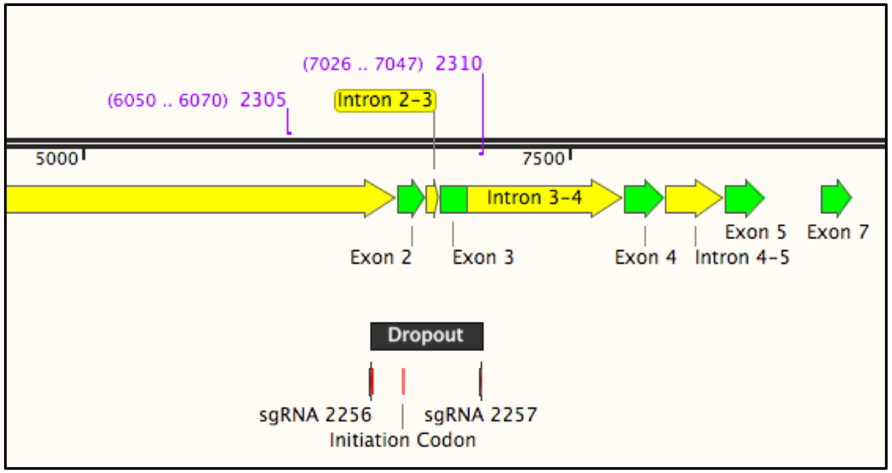

Phusion High-Fidelity PCR			
CYCLE STEP	CYCLES	TEMP	TIME
Initial denaturation	1	98°C	30 seconds
Denaturation		98°C	10 seconds
Annealing	30	72°C	30 seconds
Extension		72°C	1 minute
Final extension	1	72°C	5 minutes
Hold	1	4°C	∞

2256 sgRNA	GGAGGATCTGTGGAGTTC
2257 sgRNA	GAATCCCTGTTAGGTACC

2305 Forward	AAGGAGAGTCCTCTGCATACT
2310 Reverse	ACCTAACAGGGATTCTGTATGC

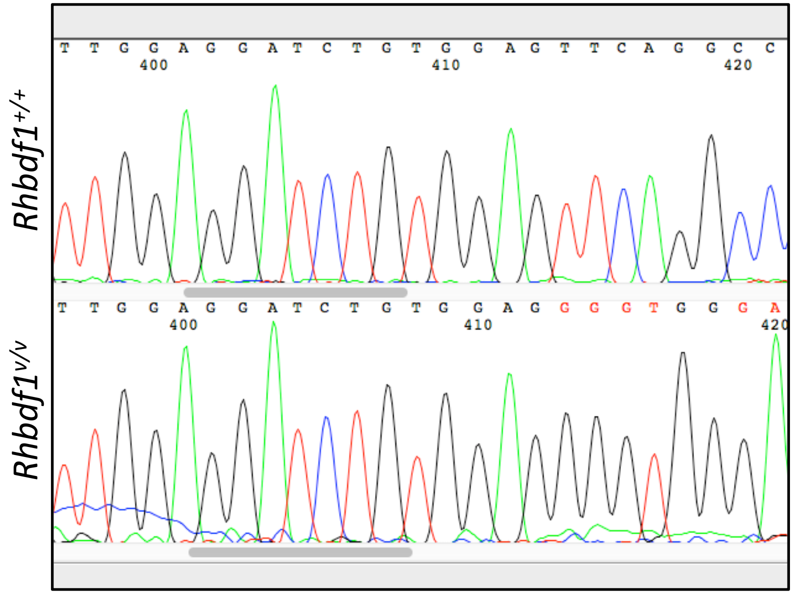

Wildtype sequence	TTGTTGGAGGATCTGTGGAGTTCAGGCCCAGAGCATGCCA
Mutant sequence	TTGTTGGAGGATCTGTGGAG-GGGTGGGAATGGGGCAAAGG

Supplementary Figure 4

a

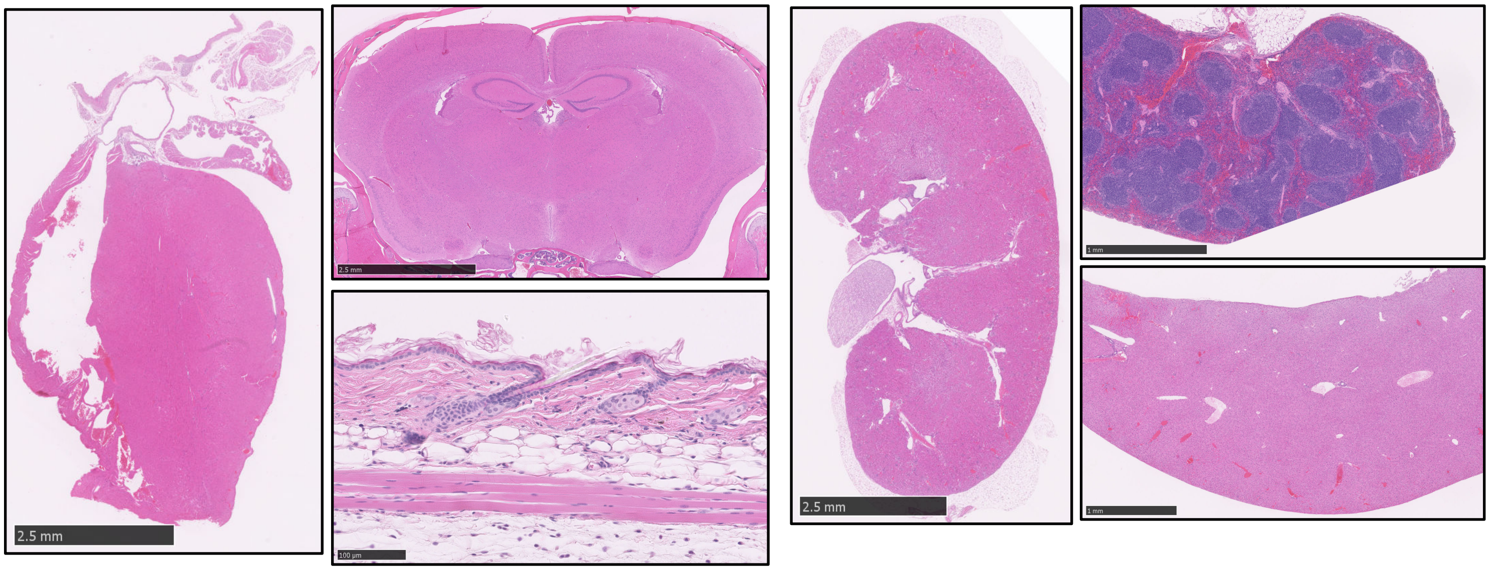

b Splice variants of *Rhbdf1*$^{v/v}$

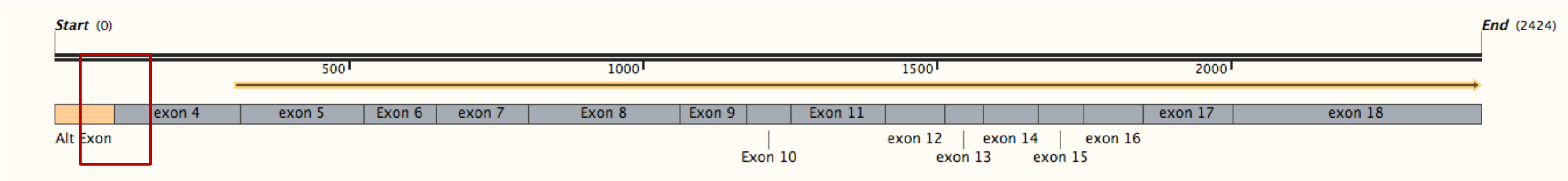

CAGGTACGCTTTGGGCGTGTTCATACTCTGCCCCTTTTGAGGCCGAGGGCTGCTCGAAAGGCCCACCCACAGCGCCAGTCTCTCTCATGGTCCTTGCTCAGGG
GGACAGCAGACTGGTTTGGAGTGAGCAAGGACAGTGACAGCACCCAGAAGTGGCAGCGCAAGAGCATCCGTCACTGCAGCCAGCGGTATGGGAAGCTAAA
GCCGCAGGTCATCCGGGAGCTGGACCTGCCCAGCCAG

Transcript variant	NCBI Reference Sequence
X1	XM_011243658.2
X2	XM_006514494.4
X3	XM_006514495.4
X4	XM_006514496.3
X9	XR_872202.2
X14	XR_001779873.2

Supplementary Figure 5

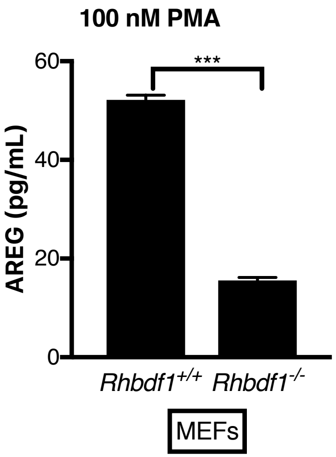

Supplementary Figure 6

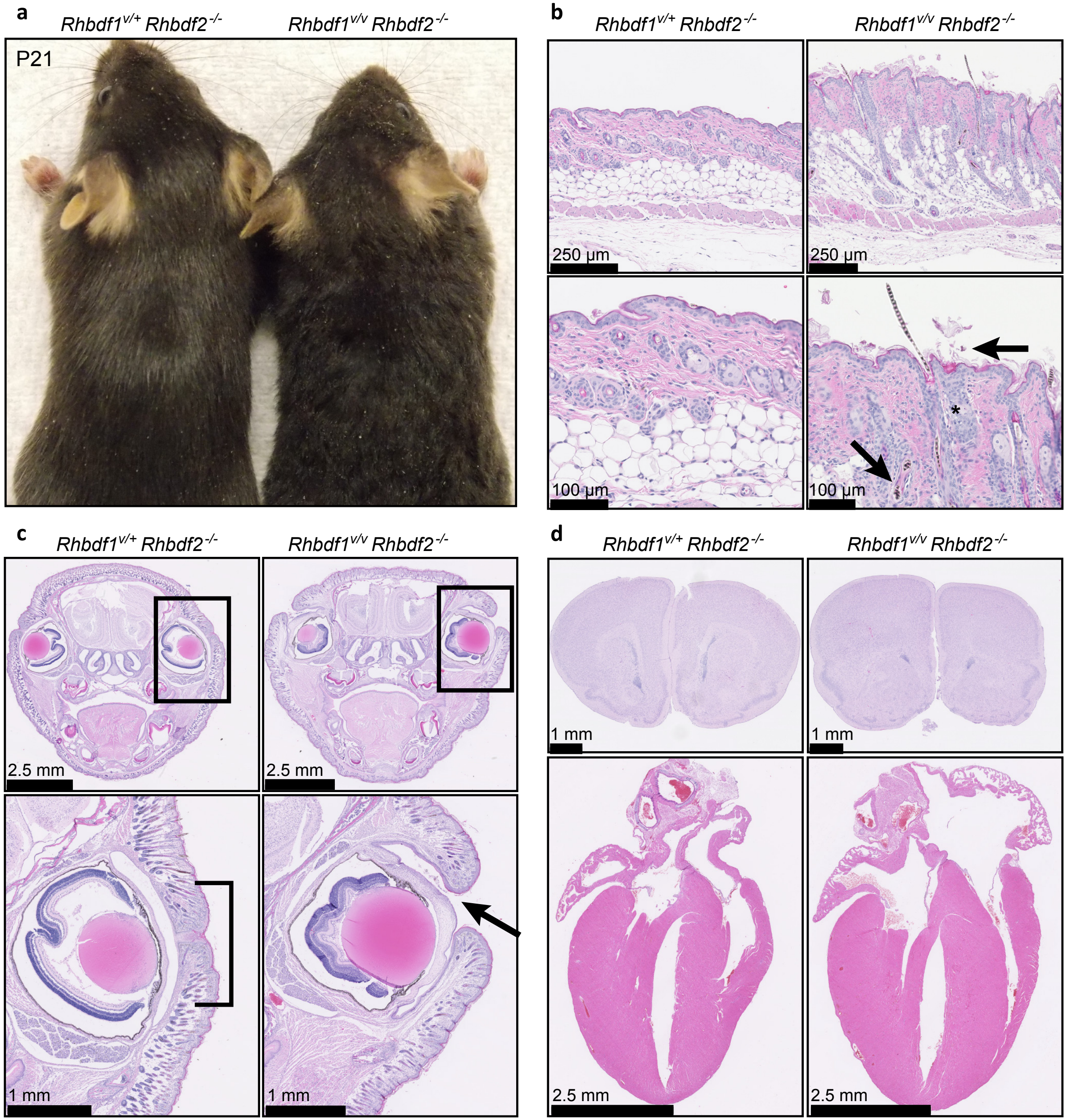

Supplementary Figure 7

a

Transcript: Mouse *Rhbdf1*-206 ENSMUST00000143988

RPCCIGTKGRCEITSREYCDFMRGYFHEEATLCSQVHCMDDVCGLLPFLNPEMTVLRDLEKLAGWHRIAIIYLLSGITGNLASAIFLPYRAEVGPAGSQFGILACLFVELFQ
SWQILARPWRAFFKLLAVVLFLFAFGLLPWIDNFAHISGFVSGLFLSFAFLPYISFGKFDLYRKRCQIIIFQVVFLGLLAGLVVLFYFYPVRCEWCEFLTCIPFTDKFCEKYELD
AQLH*

Transcript: Human *RHBDF1*-205 ENST00000448893

RPCCIGTKGRCEITSREYCDFMRGYFHEEATLCSQVHCMDDVCGLLPFLNPEMTVLRDLEKLAGWHRIAIIYLLSGVTGNLASAIFLPYRAEVGPAGSQFGILACLFVELFQ
SWQILARPWRAFFKLLAVVLFLFTFGLLPWIDNFAHISGFISGLFLSFAFLPYISFGKFDLYRKRCQIIIFQVVFLGLLAGLVVLFYVYPVRCEWCEFLTCIPFTDKFCEKYELDA
QLH*

b

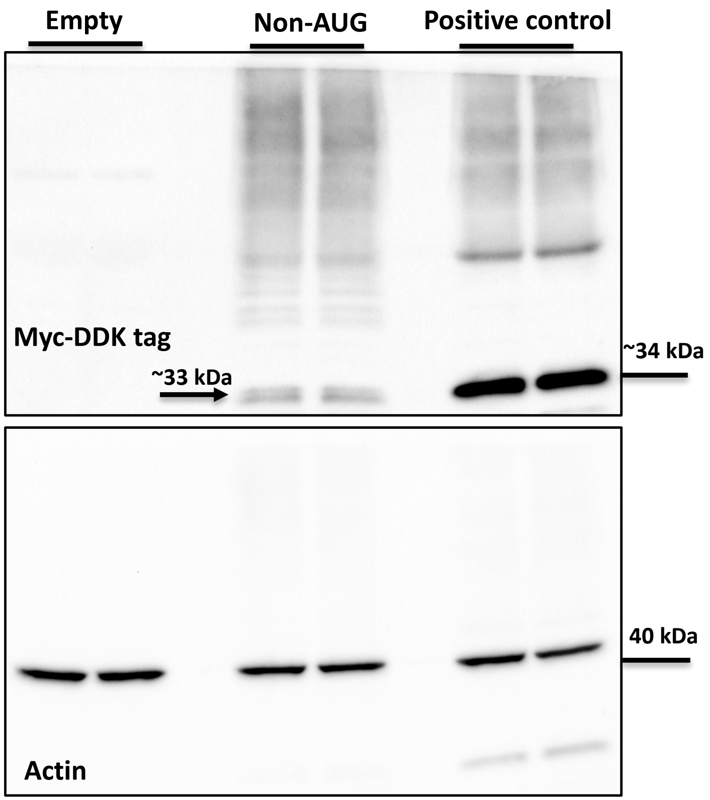

Supplementary Figure 8

a

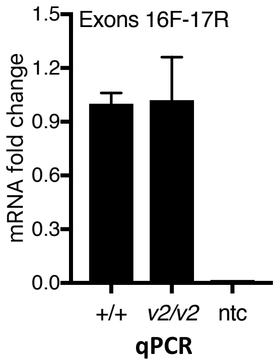

b

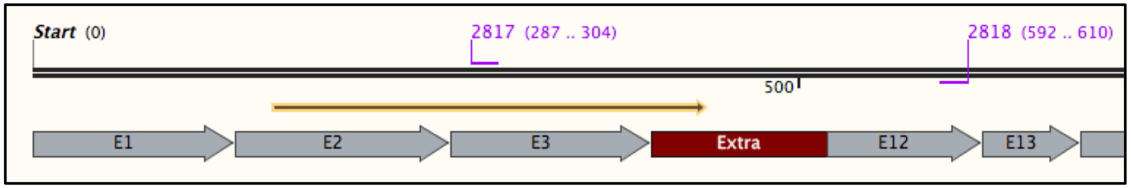

Exon 3 2817 For	AGGCTTTCTTGCGGAGTG
Exon 12 2818 Rev	GGTGGCAGACAGAACCAAA

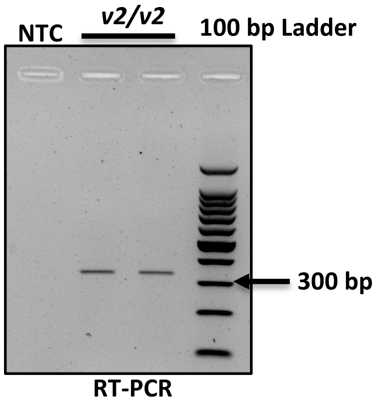

c

AGGCTTTCTTGCGGAGTGTGAGTATGCCAGCTGAGACAGCCCGGGTCCCATCACCCCACCATGAGCCTCGCCGGCTGGTGCT
GCAGCGTCAGACATCCATCACACAGACCATCCGCAGGTCCCAGGTCCCGAAAACCAAAGAAGAAGAACCCTAACAAAGAGG
ACAAGCGGCCTCGCACAGCCTTCACTGCTGAGCAGCTCCAGAGGCTCAAGGCTGAGTTTCAGACCAACAGTCCACGTTGGC
GGTGTGGGTGAAGTGGCCCGTTCATCCTAGTGCCCCAGACCTTGCAGGCAACAAGAGACAG***TTTGGTTCTGTCTGCCACC***

Bold: Primers 2817/2818

Extra: *En2* splice acceptor sequence from the targeted KO-first cassette

d

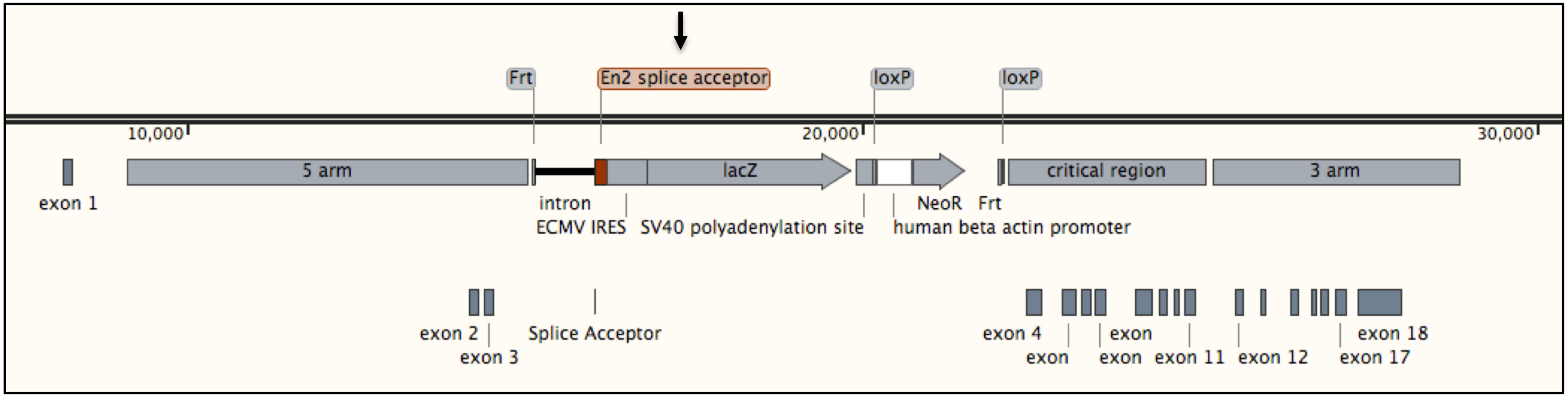

e

Putative 10.8 kDa truncated protein sequence:
MSEARRDSTSSLQRKKPPWLKLDIPAAVPPAAEEPSFLQPLRRQAFLRSVSMPAETARVPSPHHEPRRLVLQRQTSITQTIRRSQVPKTKEEEP*

Supplementary Figure 9

a

Rhbdf1 targeted KO-first allele

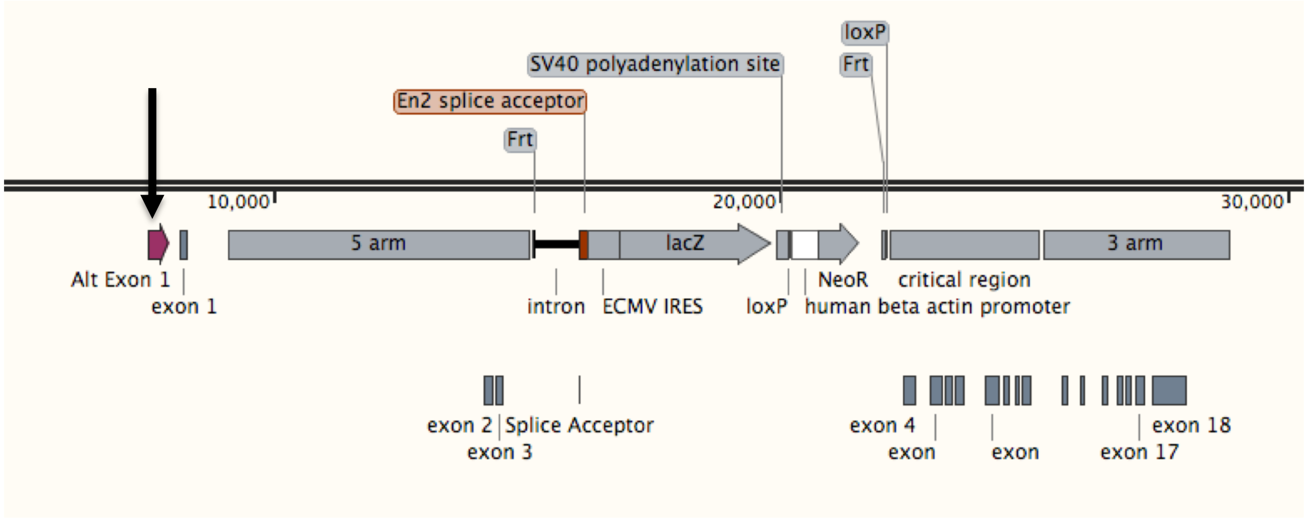

b

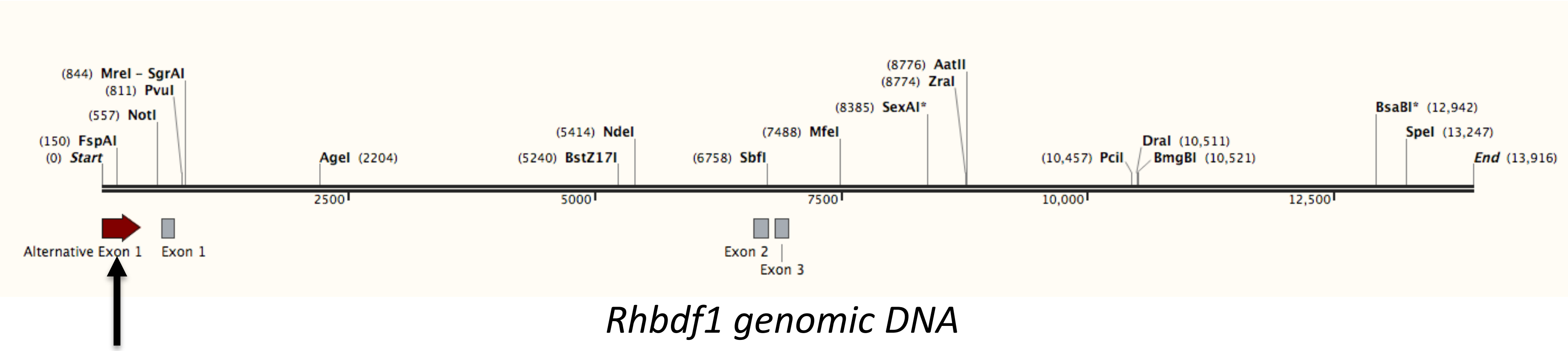

Rhbdf1 genomic DNA

<u>Alternative exon 1 sequence:</u>

tgtacctgctgggaaagaagtaggggaaggggtaattgctgagctgcggttcctgtgaaatggtgtatcagtgtatgaacgtgagtgtgcacacttataaatgtgttagggtagag
agtgtgagcccatggaagtgcattttgaataggtgtgcgcacgtgtctgtgtaaacaggtgcgagaggcctgaatgcatgaatgtttgttagaaagatcaggcgcgtgtgcacagg
acaagtgagagcttcgggaccctcagtgatgtgccagtgcgtgccttcggaaggggcgttcaatctgccttttaggtcccttcccacgccaccctcttgctttggaggtccccttcctc
tcttcccaagcacaggggcagcacaagatgaaggagtatggaaaggcgagtcca

Supplementary Figure 10

a

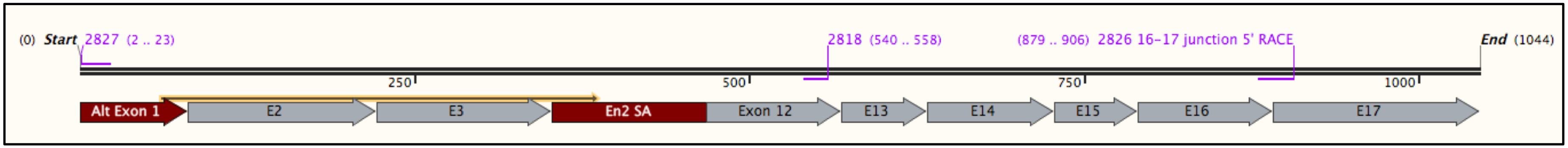

Putative 12.3 kDa truncated protein sequence:
MVYQCMNACLTAPGTMSEARRDSTSSLQRKKPPWLKLDIPAAVPPAAEEPSFLQPLRRQAFLRSVSMPAETARVPSPHHEPRRLVLQRQTSITQTIRRSQVPKTKEEEP*

b

2827 F	GTACCTGCTGGGAAAGAAGTAG
2818 R	GGTGGCAGACAGAACCAAA
2826 5' RACE	GATTACGCCAAGCTT CAAGCAGTGCAGGATCCCAGCGTGCAGG

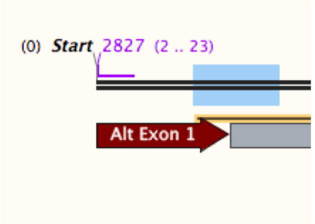

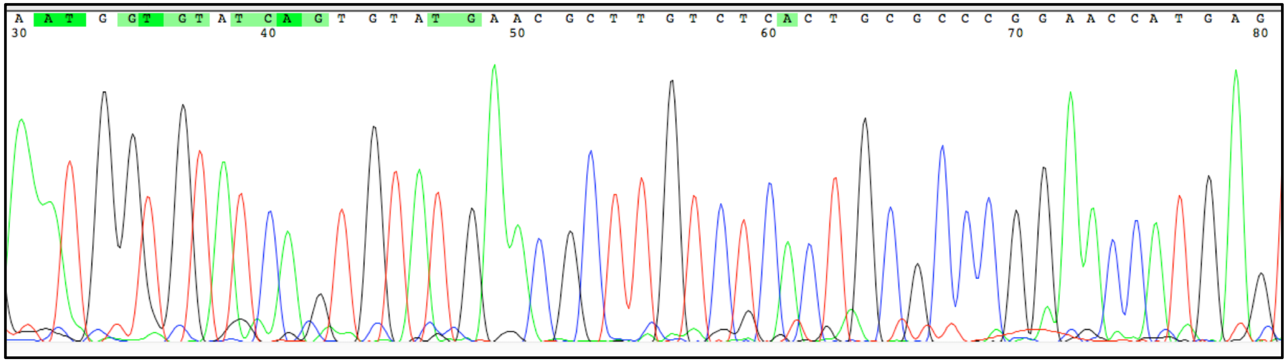

AATGGTGTATCAGTGTATGAACGCTTGTCTCACTGCGCCCGGAACCATGAG

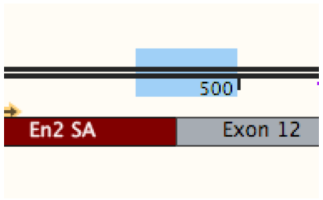

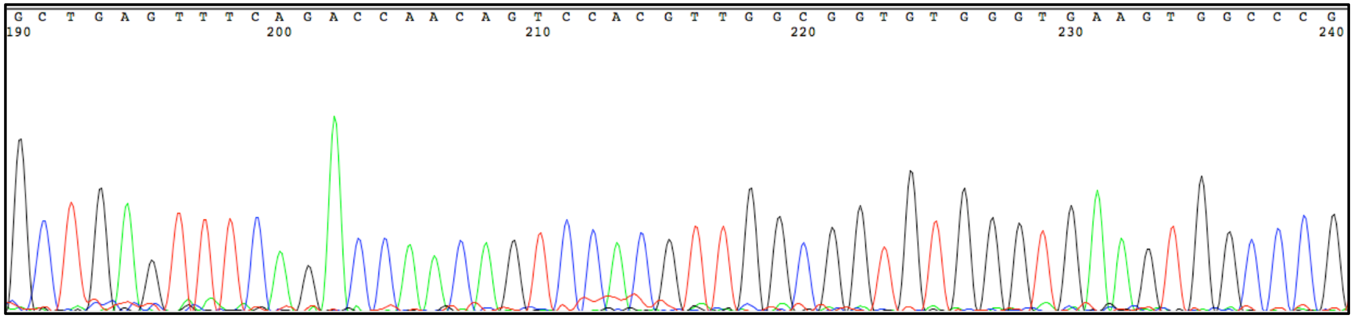

GCTGAGTTTCAGACCAACAGTCCACGTTGGCGGTGTGGGTGAAGTGGCCCG

Supplementary Figure 11

a

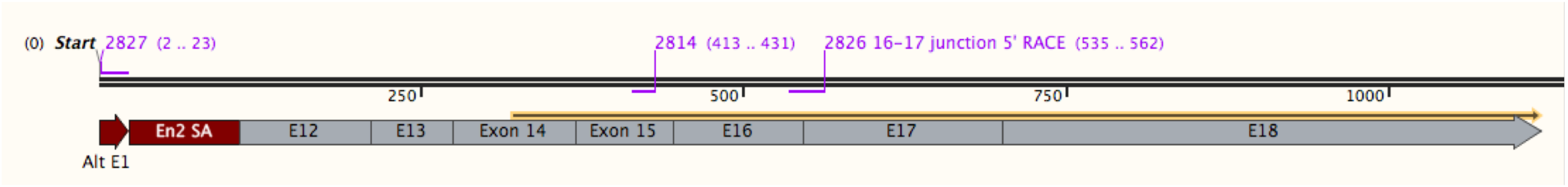

Variant 1; 32.77 kDa

MDCVITGRPCCIGTKGRCEITSREYCDFMRGYFHEEATLCSQVHCMDDVCGLLPFLNPEVPDQFYRLWLSLFLHAGILHCLVSVCFQMTVLRDLEKLAGWHRIAIIYLLSG
ITGNLASAIFLPYRAEVGPAGSQFGILACLFVELFQSWQILARPWRAFFKLLAVVLFLFAFGLLPWIDNFAHISGFVSGLFLSFAFLPYISFGKFDLYRKRCQIIIFQVVFLGLLA
GLVVLFYFYPVRCEWCEFLTCIPFTDKFCEKYELDAQLH(EQKLISEEDLDYKDDDDK)

b

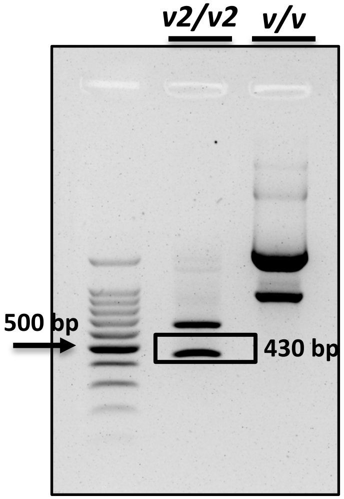

2827-2814 RT-PCR

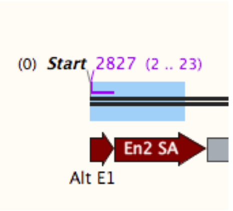

TGAGCCTCTGGAGCTGCTCAGCAGTGAAGGCTGTGCGAGGCCGCTT
GTCCTCTTTGTTAGGGTTCTACTTCTTTCCCAGCAGGTACA

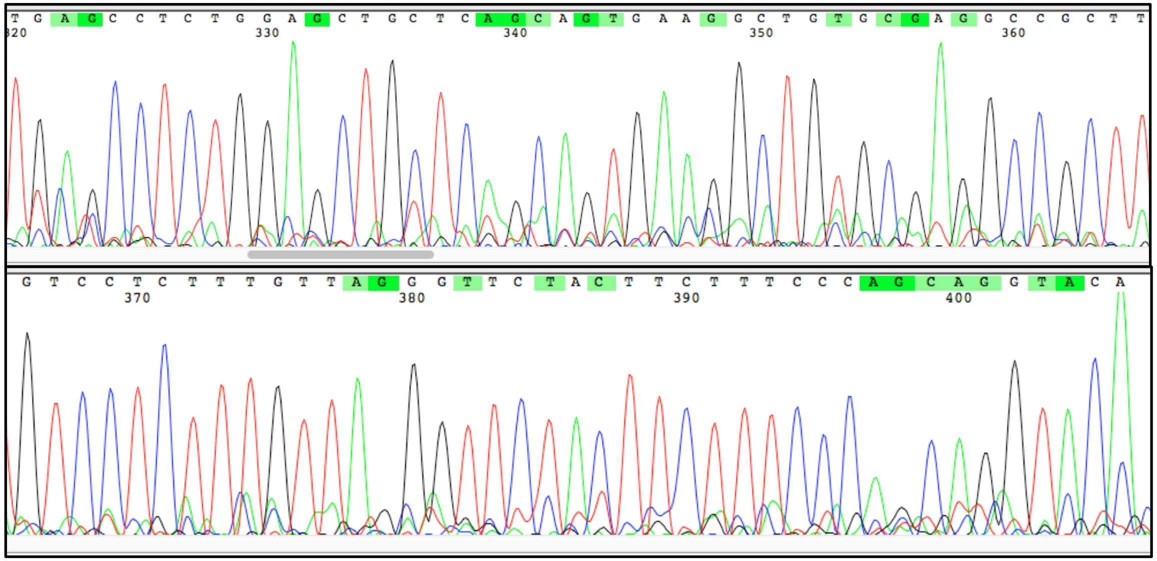

Taq polymerase PCR			
CYCLE STEP	CYCLES	TEMP	TIME
Initial denaturation	1	95°C	2 minutes
Denaturation	30	95°C	15 seconds
Annealing		52°C	30 seconds
Extension		68°C	2 minute
Final extension	1	72°C	5 minutes
Hold	1	4°C	∞

Supplementary Figure 12

a

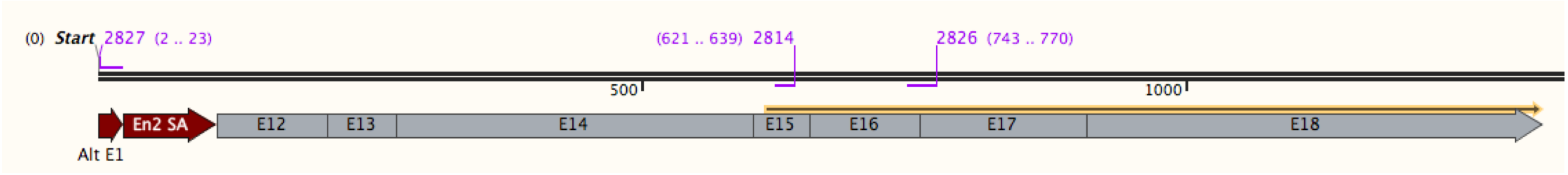

Variant 2; 29.6 kDa
MRGYFHEEATLCSQVHCMDDVCGLLPFLNPEVPDQFYRLWLSLFLHAGILHCLVSVCFQMTVLRDLEKLAGWHRIAIIYLLSGITGNLASAIFLPYRAEVGPAG
SQFGILACLFVELFQSWQILARPWRAFFKLLAVVLFLFAFGLLPWIDNFAHISGFVSGLFLSFAFLPYISFGKFDLYRKRCQIIIFQVVFLGLLAGLVVLFYFYPVRCE
WCEFLTCIPFTDKFCEKYELDAQLH(EQKLISEEDLDYKDDDDK)

b

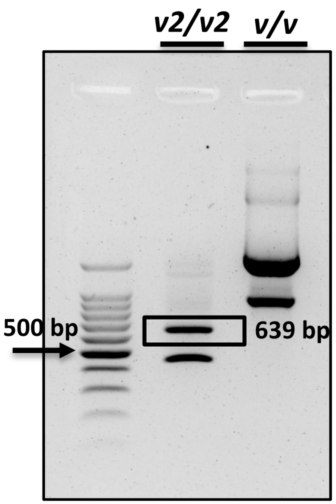

2827-2814 RT-PCR

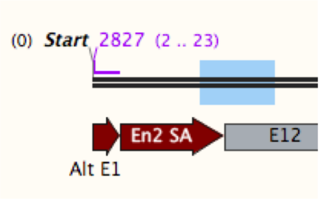

GCTGAGTTTCAGACCAACAGTCCACGTTGGCGGTGT
GGGTGAAGTGGCCCGTTCATCCTAG

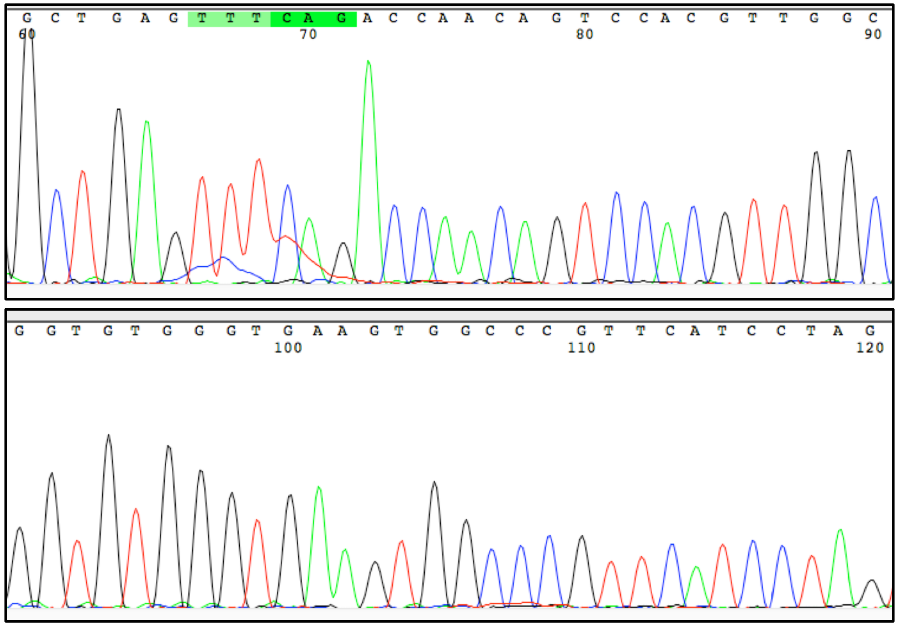

Supplementary Figure 13

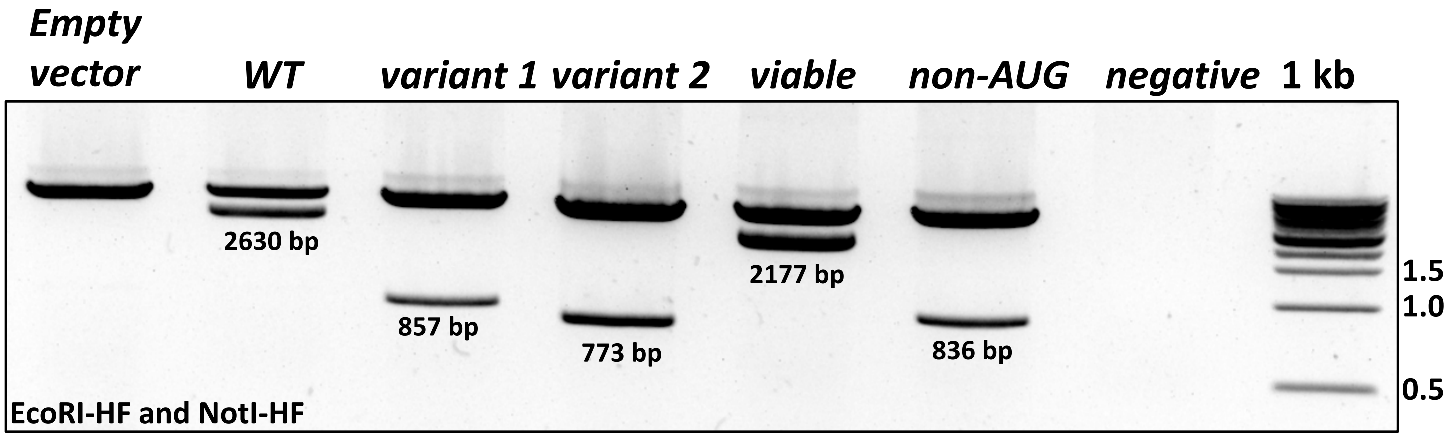

Supplementary Figure 14

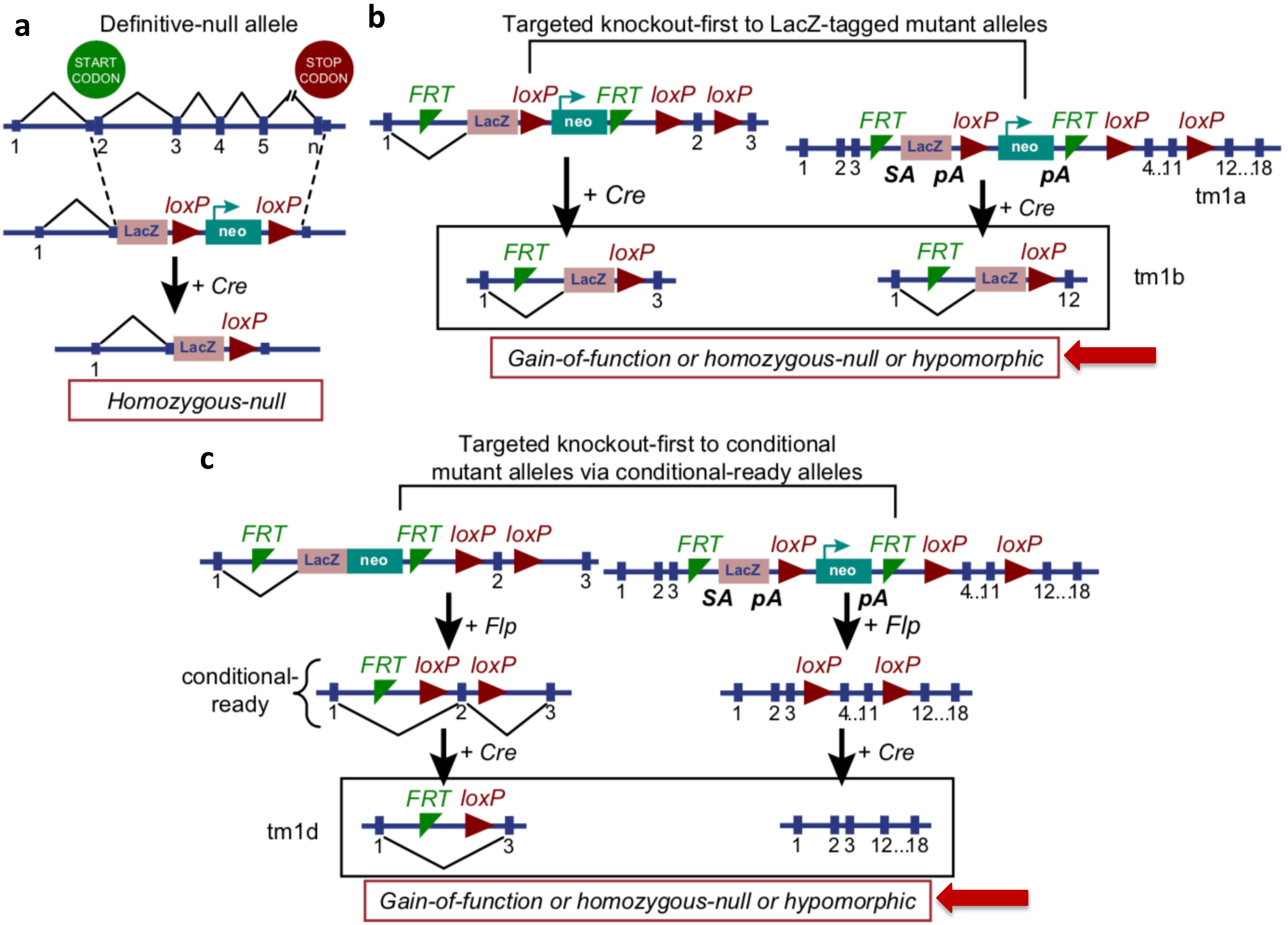

Supplementary Figure 15

a

B6N(Cg)-*Rhbdf1*$^{tm1.1(KOMP)Vlcg}$/J

Wt For	GCTCAGGTTTCAACCGACTC
Wt Rev	CAGGGAAGTCCACCATGTCT
Mt For	CGCTCCCAGTCCATTTATTG
Mt Rev	CGGTCGCTACCATTACCAGT

Taq polymerase PCR			
CYCLE STEP	CYCLES	TEMP	TIME
Initial denaturation	1	95°C	2 minutes
Denaturation	30	95°C	15 seconds
Annealing		62.5°C	30 seconds
Extension		68°C	2 minute
Final extension	1	72°C	5 minutes
Hold	1	4°C	∞

b

Rhbdf2 KOMP allele

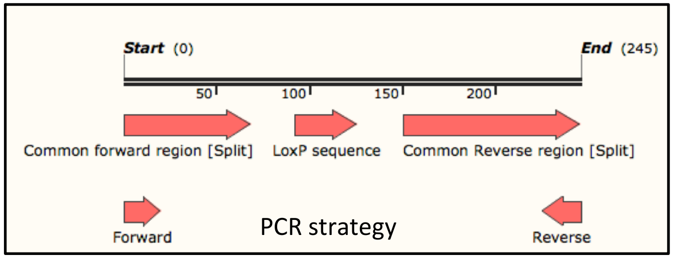

Forward	AGTAGCATCTCCCCGTGGAT
Reverse	AACCAATGGACCATGGACCC

Taq polymerase PCR			
CYCLE STEP	CYCLES	TEMP	TIME
Initial denaturation	1	95°C	2 minutes
Denaturation	30	95°C	15 seconds
Annealing		55°C	30 seconds
Extension		68°C	2 minute
Final extension	1	72°C	5 minutes
Hold	1	4°C	∞

Wildtype

Heterozygous

Homozygous

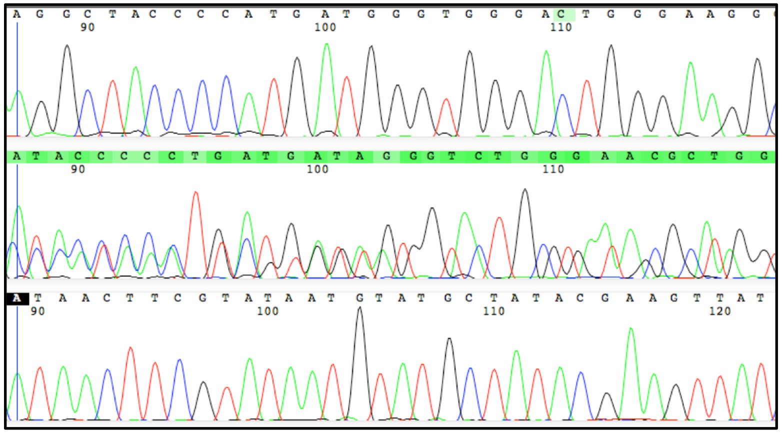

Supplement: Supplementary file 1 — Additional file 1: Figure S1. Gene-targeting strategies for generating null or conditional mutant alleles: a. In the definitive-null design, the complete coding sequence of a gene is flanked by two loxP sites, and recombination with a cre recombinase excises the floxed gene segment, creating LacZ-tagged null alleles. Loss of the complete coding sequence of the target gene ensures homozygous-null alleles. b. Targeted KO-first alleles harbor FRT sites flanking “LacZ and a neo gene,” and also harbor loxP sites flanking either an exon (exon 2, left) or specific exons chosen to be excised (exons 4 through 11, right, in this case), permitting recombination with Flp or cre recombinases and enabling conditional mutagenesis. Application of cre recombinase deletes exons flanked by two loxP sites, generating LacZ-tagged null alleles (tm1b). Application of Flp recombinase to this allele is described in the legend for 1C. c. Application of Flp recombinase to targeted KO-first alleles reverts the knockout-first alleles into conditional-ready alleles by excising lacZ and a neo gene. Subsequent recombination of conditional-ready alleles with cre recombinase generates conditional mutant alleles (tm1d). Figure S2. Sub-viability in Rhbdf1-/-Rhbdf2-/- double mutant mice (A6): a. Breeding strategy and the number of mice generated and genotyped by PCR b & c. Rhbdf1-/-Rhbdf2-/- double mutant mice displayed “eyelids open at birth” phenotype (arrows) in mouse embryos harvested on embryonic day 15 (e15.5) (B) and in pups at day 2 (C). No defects were observed in Rhbdf1+/-Rhbdf2-/- littermates. Figure S3. PCR and genotyping of Rhbdf1v/v allele: Schematic showing the TRU-guides (sgRNAs 2256 and 2257) used to excise exons 2 and 3 in the Rhbdf1 gene. Also shown are the PCR primers and Taq DNA Polymerase thermocycling conditions used to genotype Rhbdf1v/v mice (A7). PCR products were sequenced to verify the 569-bp deletion. Figure S4. Histology and 5’ RACE products of Rhbdf1v/v allele: a. H&E sec [file 13059_2020_2086_MOESM1_ESM.pdf]
