# Supplementary material for: Genes adapt to outsmart gene-targeting strategies in mutant mouse strains by skipping exons to reinitiate transcription and translation
Source: Genome Biol. 2020 Jul 9;21:168. doi: 10.1186/s13059-020-02086-0 (PMC7350591; doi:10.1186/s13059-020-02086-0)
Supplement: Supplementary file 6 — Additional file 6. Table S5. Genes retaining residual protein expression upon CRISPR/Cas9-induced indels. [file 13059_2020_2086_MOESM6_ESM.pdf]

Species	Gene(s)	5' Incomplete	3' Incomplete	Approach	Reference
Human	*FLOT1*	5’	3'	CRISPR/Cas9	[1]
Mouse	*Gli3*	-	3'	CRISPR/Cas9	[2]
Human	*TEAD1*	5'	3'	Non-AUG start	[3]
Human	*MRVI1*	5'	3'	Non-AUG start	[3]
Human	*PIM1*	5'	3'	Non-AUG start	[3]
Human	*EIF4G2*	5'	3'	Non-AUG start	[3]
Human	*MYC*	-	3'	Non-AUG start	[3]
Human	*RARB*	-	3'	Non-AUG start	[3]
Human	*TEAD4*	5'	3'	Non-AUG start	[3]
Human	*R3HCC1*	5'	3'	Non-AUG start	[3]
Mouse	*Ctnnb1*	5'	3'	CRISPR/Cas9	[4]
Human	*LMNA*	5'	3'	CRISPR/Cas9	[4]
Zebrafish	*cd36*	5'	3'	ENU	[5]
Zebrafish	*pla2g12b*	5'	-	ENU	[5]
Zebrafish	*smyd1*	5'	-	CRISPR/Cas9	[5]
Human	*PHACTR1*	5'	3'	CRISPR/Cas9	[6]
Human	*CTNNB1*	5'	3'	CRISPR/Cas9	[7]
Human	*AXIN1*	5'	3'	CRISPR/Cas9	[7]
Human	*LRP5*	5'	-	CRISPR/Cas9	[7]
Human	*LRP6*	5'	3'	CRISPR/Cas9	[7]
Human	*PTEN*	5'	-	CRISPR/Cas9	[7]
Human	*TBK1*	-	3'	CRISPR/Cas9	[7]
Human	*BAP1*	5'	-	CRISPR/Cas9	[7]
Human	*TLE3*	5'	3'	CRISPR/Cas9	[7]
Human	*PPM1A*	-	3'	CRISPR/Cas9	[7]
Human	*BCL2L2*	5'	3'	CRISPR/Cas9	[7]
Human	*NGLY1*	5'	3'	CRISPR/Cas9	[8]
Human	*PI4KB*	5'	3'	CRISPR/Cas9	[8]
Human	*BRD4*	5'	3'	CRISPR/Cas9	[8]
Human	*BRD3*	-	3'	CRISPR/Cas9	[8]
Human	*MTF2*	5'	3'	CRISPR/Cas9	[8]
Human	*BAZ2A*	5'	3'	CRISPR/Cas9	[8]

References:

1. Kapahnke M, Banning A, Tikkanen R: **Random Splicing of Several Exons Caused by a Single Base Change in the Target Exon of CRISPR/Cas9 Mediated Gene Knockout.** *Cells* 2016, **5**.
2. Makino S, Fukumura R, Gondo Y: **Illegitimate translation causes unexpected gene expression from on-target out-of-frame alleles created by CRISPR-Cas9.** *Sci Rep* 2016, **6:**39608.
3. Diaz de Arce AJ, Noderer WL, Wang CL: **Complete motif analysis of sequence requirements for translation initiation at non-AUG start codons.** *Nucleic Acids Res* 2018, **46:**985-994.
4. Mou H, Smith JL, Peng L, Yin H, Moore J, Zhang XO, Song CQ, Sheel A, Wu Q, Ozata DM, et al: **CRISPR/Cas9-mediated genome editing induces exon skipping by alternative splicing or exon deletion.** *Genome Biol* 2017, **18:**108.
5. Anderson JL, Mulligan TS, Shen MC, Wang H, Scahill CM, Tan FJ, Du SJ, Busch-Nentwich EM, Farber SA: **mRNA processing in mutant zebrafish lines generated by chemical and CRISPR-mediated mutagenesis produces unexpected transcripts that escape nonsense-mediated decay.** *PLoS Genet* 2017, **13:**e1007105.
6. Lalonde S, Stone OA, Lessard S, Lavertu A, Desjardins J, Beaudoin M, Rivas M, Stainier DYR, Lettre G: **Frameshift indels introduced by genome editing can lead to in-frame exon skipping.** *PLoS One* 2017, **12:**e0178700.
7. Tuladhar R, Yeu Y, Tyler Piazza J, Tan Z, Rene Clemenceau J, Wu X, Barrett Q, Herbert J, Mathews DH, Kim J, et al: **CRISPR-Cas9-based mutagenesis frequently provokes on-target mRNA misregulation.** *Nat Commun* 2019, **10:**4056.
8. Smits AH, Ziebell F, Joberty G, Zinn N, Mueller WF, Clauder-Munster S, Eberhard D, Falth Savitski M, Grandi P, Jakob P, et al: **Biological plasticity rescues target activity in CRISPR knock outs.** *Nat Methods* 2019, **16:**1087-1093.
