# Supplementary material for: Genes adapt to outsmart gene-targeting strategies in mutant mouse strains by skipping exons to reinitiate transcription and translation
Source: Genome Biol. 2020 Jul 9;21:168. doi: 10.1186/s13059-020-02086-0 (PMC7350591; doi:10.1186/s13059-020-02086-0)
Supplement: Supplementary file 9 — Additional file 9. Review history. [file 13059_2020_2086_MOESM9_ESM.docx]

Review History

**First round of review**

**Reviewer 1**

**Comments to author:**

Vishnu et al performed the gene-disruption in mice with diverse strategies that could affect the phenotype of resulting mutant mice. To investigate the difference, the authors analyzed several Rhbdf-1 deficient mice and concluded that the difference is due to exon skipp­­­ing or non-AUG initiation generated novel transcripts, which could to some extent rescue the null pheno­­­type. However, the experiments could not completely achieve the conclusion. The concerns for this manuscript are shown in the following.
­­­
The rescue experiments were finished in MEF cell in vitro, the authors should design an experiment to rescue the null phenotype in vivo.
The expression of other unaffected isoforms may be upregulated to rescue the phenotype induced by knocking out one isoform. Then the author should perform experiments to differentiate the protein isoforms and the newly generated ­truncated transcripts. Are the other protein isoforms or the new truncated transcripts could partially rescue the null phenotype? To avoid isoforms, it is better to perform the experiments targeting the gene encoding a single isoform.
In the analysis, the author did the bioinformatics to get the percentage of the 5' incomplete CDS or 3' incomplete CDS. The author should define incomplete CDS. How to tell the difference between the multiple isoforms from incomplete CDS?
Fig4g, the basal AREG level in Rhbdf1+/+ MEFs and Rhbdf1-/- MEFs seem similar, but look different from the value shown SupFig5.

Minor:
……AREG levels following stimulation with PMA (Fig 3l). Fig 3l is not correct.

**Reviewer 2**

**Comments to author:**

This manuscript from Hosur et al is an in-depth study of induced mutations of the Rhbdf1 gene.  Based on conflicting phenotypes published by different groups in the literature, they show that deletion of presumed "critical exons" including the most 5' translation start site still allow for a truncated and stable mRNA and protein that is at least partially functional.  More surprisingly, a much larger internal deletion had similar results.  Bottom line: a presumed null is not necessarily a null unless one can prove that there is no residual protein produced that has function.

This is a very nice paper that should garner some attention. It is a wake-up call to those who are not careful in thoroughly designing or analyzing alleles made by any method.  However, it is not a new finding; geneticists are aware of such possibilities…at least the careful ones.  

There are no major flaws in this study, and the paper is relatively clear.  I have just a few issues and suggestions, bulleted below.


* Just because deleting "critical exons" can still result in truncated transcripts with a downstream AUG, that doesn't mean that the truncated transcript or protein can actually provide function; thus, the estimates of numbers of genes that might be inaccurately judged from "knockout" allelels is probably exaggerated substantially.  I suggest qualifying these claims, especially in the Abstract.  For proteins with known domains, it might be suggested that these be judged as "critical exons."  The authors may also want to make suggestions regarding whether targeting the TSS for deletion might be desireable.

* p. 3 lines 42-47.  Please elaborate more on why "It seems unlikely" and "It seems more likely."

* first line page 4.  It is not mentioned earlier in the para that Rhbdf2 mice are also being made. In fact, the source or creation of these mice is not mentioned anywhere in the main text, including not in the Methods.  How is it known that this is a null?  

* The authors refer to exons 2 and 3 of Rhbdf1 as "critical." Who defined them as such?  Obviously, they are not.

**Authors Response**

**Point-by-point responses to the reviewers’ comments:**

*We thank the referees for their favorable comments, and below we address their concerns on a point-by-point basis.*

Reviewer reports:

Reviewer #1:

Vishnu et al performed the gene-disruption in mice with diverse strategies that could affect the phenotype of resulting mutant mice. To investigate the difference, the authors analyzed several Rhbdf-1 deficient mice and concluded that the difference is due to exon skipp­­­ing or non-AUG initiation generated novel transcripts, which could to some extent rescue the null pheno­­­type. However, the experiments could not completely achieve the conclusion. The concerns for this manuscript are shown in the following.

The rescue experiments were finished in MEF cell in vitro, the authors should design an experiment to rescue the null phenotype in vivo.

*We apologize for not highlighting the significance of in vivo rescue experiments in the main text. In supplemental figures 2a, 2b, and 2c, we show that whereas the Rhbdf1 null allele in combination with the Rhbdf2 null allele, results in sub-viability, the Rhbdf1 viable allele in combination with the Rhbdf2 null allele results in healthy, viable, and fertile mice (supplemental figures 6a, 6b,6c, and 6d), demonstrating in vivo rescue of sub-viability, owing to RHBDF1 deficiency, by RHBDF1 truncated isoforms.*

*Additionally, our unpublished findings (subject of another manuscript) also demonstrate strong evidence for rescue of the sub-viable phenotypes:*

a. *Rhbdf1-/- Rhbdf2-/- (sub-viable) versus Rhbdf1v/v Rhbdf2-/- (viable) (Present study)*
b. *Rhbdf1-/- Rhbdf2cub/cub (sub-viable; mice die within two weeks; arrow) versus Rhbdf1v/v Rhbdf2cub/cub (viable, fertile, and healthy; arrowhead) (unpublished findings).*

c. *Rhbdf1-/- Rhbdf2 P159L/P159L (sub-viable) versus Rhbdf1v/v Rhbdf2 P159L/P159L (viable) (unpublished findings).*

The expression of other unaffected isoforms may be upregulated to rescue the phenotype induced by knocking out one isoform. Then the author should perform experiments to differentiate the protein isoforms and the newly generated ­truncated transcripts. Are the other protein isoforms or the new truncated transcripts could partially rescue the null phenotype?

*This is a great suggestion and it is likely that unaffected isoforms could be upregulated. In the present study, however, we have observed no significant differences in the transcript levels of isoforms generated between wildtype and Rhbdf1 viable-2 homozygous mice when examined by quantitative real-time PCR (Supplemental Figure 8a). We further demonstrate using functional assays that all three truncated protein forms— variant 1, variant 2, and the non-AUG variant—are equally effective at regulating stimulated-amphiregulin secretion in Rhbdf1 null mouse embryonic fibroblasts.*

To avoid isoforms, it is better to perform the experiments targeting the gene encoding a single isoform.

*This is again a great suggestion. However, since transcription and translation re-initiation can occur in unexpected ways, it is not possible to accurately predict which genes encoding single isoforms are predisposed to illegitimate transcription and translation. We show three examples of single-isoform genes (Table 1), with no evidence of incomplete 5' or 3' coding sequences, that yield conflicting phenotypes in mice:*

*1	Caskin1 (CASK interacting protein 1): em1 (homozygous viable) and tm1b (homozygous subviable)*

*2	Dppa1 (Developmental pluripotency associated 1): tm1a (homozygous viable) and tm1b (homozygous lethal)*

*3	Zranb1 (zinc finger, RAN-binding domain containing 1): tm1a (homozygous viable) and tm1b (homozygous subviable).*

In the analysis, the author did the bioinformatics to get the percentage of the 5' incomplete CDS or 3' incomplete CDS. The author should define incomplete CDS. How to tell the difference between the multiple isoforms from incomplete CDS?

*We thank the reviewer for this comment. We now mention how we define incomplete CDS on page 24 (highlighted). Also, see below:*

*3' incomplete CDS—A protein-coding transcript which is missing the stop codon due to incomplete evidence. 5' incomplete CDS—A protein-coding transcript which is missing the start codon due to incomplete evidence.*

Fig. 4g, the basal AREG level in Rhbdf1+/+ MEFs and Rhbdf1-/- MEFs seem similar, but look different from the value shown SupFig5.

*We and others have demonstrated that RHBDF1 regulates stimulated secretion, but not the basal levels of AREG. Hence there is no significant difference in the basal levels of AREG between Rhbdf1+/+ and Rhbdf1-/- MEFs. Moreover, in Supplemental Figure 5, we only showed PMA-stimulated secretion in Rhbdf1+/+ MEFs in comparison with Rhbdf1-/- MEFs.*

Minor: AREG levels following stimulation with PMA (Fig 3l). Fig 3l is not correct.

*We thank the reviewer for this comment, we have now corrected Fig 3I to 3m.*

Reviewer #2:

This manuscript from Hosur et al is an in-depth study of induced mutations of the Rhbdf1 gene.  Based on conflicting phenotypes published by different groups in the literature, they show that deletion of presumed "critical exons" including the most 5' translation start site still allow for a truncated and stable mRNA and protein that is at least partially functional.  More surprisingly, a much larger internal deletion had similar results.  Bottom line: a presumed null is not necessarily a null unless one can prove that there is no residual protein produced that has function.

This is a very nice paper that should garner some attention. It is a wake-up call to those who are not careful in thoroughly designing or analyzing alleles made by any method.  However, it is not a new finding; geneticists are aware of such possibilities…at least the careful ones.

There are no major flaws in this study, and the paper is relatively clear.  I have just a few issues and suggestions, bulleted below.

*We thank the reviewer for positive comments.*

* Just because deleting "critical exons" can still result in truncated transcripts with a downstream AUG, that doesn't mean that the truncated transcript or protein can actually provide function; thus, the estimates of numbers of genes that might be inaccurately judged from "knockout" alleles is probably exaggerated substantially.

*We apologize for not clearly defining “critical exons” in the manuscript. Exons 2 and 3 have been considered “critical exons” because the start codon is located in exon 2 of the Rhbdf1 gene. Additionally, our intention with the CRISPR/Cas9 gene editing allele was to generate mice that could mimic KO-first mice generated by Li et al. which were viable, despite lacking exons 4-11.*

*Regarding the estimates, we strongly believe that the number of genes (4%) could be a conservative estimate because in the present study we only considered mice showing severe (subviable or lethal versus viable), with conflicting phenotypes (table 1). It is highly likely that among the other strains generated using the KO-first strategy that are viable, there could be strains that are gain-of-function alleles rather than the null alleles, however the phenotype could be masked until challenged, for instance with a stimulant, or in combination with additional genetic deficiencies. Moreover, the observation that the KO-first allele (tm1a) generates viable mice, whereas the same allele after cre-mediated recombination (tm1b) generates homozygous lethal mice, clearly demonstrates that the residual transcripts and proteins are functional (table 1).*

I suggest qualifying these claims, especially in the Abstract.  For proteins with known domains, it might be suggested that these be judged as "critical exons."  The authors may also want to make suggestions regarding whether targeting the TSS for deletion might be desirable.

*This is a great suggestion. Over the years, the term “critical exons” has been defined differently by different groups;*

*Wolfgang Wurst and Ralf Kuhn et al. define the critical exon as “the first exon downstream of the gene’s exon 1 that exhibits different reading frames upon splicing to its preceding and its following exon [1].”*

*International Mouse Phenotyping Consortium defines critical exon as “an exon common to all transcript variants that, when deleted, creates a frame-shift mutation.”*

*We believe the region to be excised or targeted could be locus dependent, and our goal with this paper is to warn researchers to thoroughly test for any residual activity, unless most of the exons are deleted.*

*We have now replaced the term critical exon with ‘an exon(s) carrying the start codon in the Rhbdf1 gene’ in the revised manuscript.*

* p. 3 lines 42-47.  Please elaborate more on why "It seems unlikely" and "It seems more likely."

*We have now added the following text to the manuscript:*

*It seems unlikely that there is a modifier in the B6 congenic E2a-Cre strain [B6.FVB-TgEIIa-cre; Stock No: 003724] that could account for the difference in the phenotype observed in the single and double mutant mice generated on the C57BL/6J background by Christova et al. It seems more likely, rather, that the differences in the phenotypes could be due to effects of the different gene targeting strategies employed (Fig. 1a) by the two studies to generate Rhbdf1 KO mice. Notably, both the transcription start site and the start codon remain unaltered by the targeted KO-first strategy.*

* first line page 4.  It is not mentioned earlier in the para that Rhbdf2 mice are also being made. In fact, the source or creation of these mice is not mentioned anywhere in the main text, including not in the Methods.

*We have now added the following statement in the methods section:*

*Generation of Rhbdf2-/- mice has been described previously [2].*

How is it known that this is a null?

*This is a good question. Several groups have generated Rhbdf2-/- mice using different strategies. Although wildtype are indistinguishable from the null mice, phenotypically, Rhbdf2-/- mice show reduced stimulated-secretion of epidermal growth factor ligands and tumor necrosis factor [2]. Additionally, the Lenny Shultz lab and the Bruce Beutler lab [3] have separately performed complementation tests with mutant alleles of Rhbdf2 (Rhbdf2cub and Rhbdf2sin, respectively), confirming genetic non-complementation, and that the Rhbdf2 KO mice are null.*

* The authors refer to exons 2 and 3 of Rhbdf1 as "critical." Who defined them as such?  Obviously, they are not.

*This is a great suggestion. We have now replaced the term critical exon with ‘the exon(s) carrying the start codon’ in the revised manuscript.*

*References:*

*1.	Friedel RH, Wurst W, Wefers B, Kuhn R: Generating conditional knockout mice. Methods Mol Biol 2011, 693:205-231.*

*2.	Hosur V, Johnson KR, Burzenski LM, Stearns TM, Maser RS, Shultz LD: Rhbdf2 mutations increase its protein stability and drive EGFR hyperactivation through enhanced secretion of amphiregulin. Proc Natl Acad Sci U S A 2014, 111:E2200-2209.*

*3.	Siggs OM, Xiao N, Wang Y, Shi H, Tomisato W, Li X, Xia Y, Beutler B: iRhom2 is required for the secretion of mouse TNFα. Blood 2012, 119:5769-5771.*

**Second round of review**

**Reviewer 1**

The authors have addressed all of my concerns. Thanks.

**Reviewer 2**

No more questions on the ms.
